# Improving Visual Defect Detection and Localization in Industrial Thermal Images Using Autoencoders

**DOI:** 10.3390/jimaging9070137

**Published:** 2023-07-07

**Authors:** Sasha Behrouzi, Marcel Dix, Fatemeh Karampanah, Omer Ates, Nissy Sasidharan, Swati Chandna, Binh Vu

**Affiliations:** 1 Applied Data Science and Analytics, SRH University, 69123 Heidelberg, Germany; fatemeh.karampanah@stud.hochschule-heidelberg.de (F.K.); nissy.sasidharan@stud.hochschule-heidelberg.de (N.S.); swati.chandna@srh.de (S.C.); binh.vu@srh.de (B.V.); 2 Industrial Data Analytics, ABB Corporate Research, 68526 Ladenburg, Germany

**Keywords:** anomaly detection, deep learning, novelty detection, autoencoder, industrial image, thermal image

## Abstract

Reliable functionality in anomaly detection in thermal image datasets is crucial for defect detection of industrial products. Nevertheless, achieving reliable functionality is challenging, especially when datasets are image sequences captured during equipment runtime with a smooth transition from healthy to defective images. This causes contamination of healthy training data with defective samples. Anomaly detection methods based on autoencoders are susceptible to a slight violation of a clean training dataset and lead to challenging threshold determination for sample classification. This paper indicates that combining anomaly scores leads to better threshold determination that effectively separates healthy and defective data. Our research results show that our approach helps to overcome these challenges. The autoencoder models in our research are trained with healthy images optimizing two loss functions: mean squared error (MSE) and structural similarity index measure (SSIM). Anomaly score outputs are used for classification. Three anomaly scores are applied: MSE, SSIM, and kernel density estimation (KDE). The proposed method is trained and tested on the 32 × 32-sized thermal images, including one contaminated dataset. The model achieved the following average accuracies across the datasets: MSE, 95.33%; SSIM, 88.37%; and KDE, 92.81%. Using a combination of anomaly scores could assist in solving a low classification accuracy. The use of KDE improves performance when healthy training data are contaminated. The MSE+ and SSIM+ methods, as well as two parameters to control quantitative anomaly localization using SSIM, are introduced.

## 1. Introduction

In today’s modern industrial landscape in which the emergence of artificial intelligence holds the potential to deliver enhanced productivity in industrial practices [1], the reliability of defect detection and condition monitoring for industrial equipment is of utmost importance [2]. By harnessing the power of machine learning algorithms to analyze thermal images, maintenance professionals can significantly enhance this reliability. This emphasizes the crucial importance of incorporating these algorithms into maintenance practices to effectively address the challenges posed by modern hybrid physical–digital systems in the industry [3]. Reliable functionality in detecting defective areas in a given set of thermal images is crucial for the maintenance of industrial equipment and products. Traditionally defective equipment parts can be detected via temperature observation. Electrical faults change the electrical resistance of the equipment, which in turn increases the temperature. This makes the use of infrared thermography cameras to spot defective areas in the industrial equipment possible by comparing the current temperature of the equipment with temperatures in reference to healthy equipment images. Manual image comparisons are time-consuming and unreliable, so several algorithms exist to detect thermal anomalies and to spot thermal differences in these images [4]. One approach is visual anomaly detection, which is a machine-learning task and can be utilized for this purpose.

Anomaly detection is a machine learning problem in which datasets are heavily biased in favor of normal classes because the abnormal class is too small. Meanwhile, detecting unseen anomaly cases makes utilizing supervised methods impossible [5]. These challenges lead researchers to lean towards methods that infer latent spaces. Autoencoders lie at the heart of these methods. They compress the input image to latent codings and subsequently reconstruct it. Anomaly scores can be calculated by studying the latent vector or reconstruction loss approaches.

Using industrial thermal images, our research comprises a training autoencoder with two different loss functions—mean squared error (MSE) and structural similarity index measure (SSIM)—and finds anomalies within the test phase by applying three anomaly scores—MSE, SSIM, and kernel density estimation (KDE). This paper uses the term anomaly score denoted by *A*(*x*). A larger anomaly score, *A*(*x*), suggests that there are possible anomalies in the test image. We group all approaches above, give their ideas, and report the survey results using classification measures. While autoencoders and alternative metrics have been extensively studied, this paper introduces a distinctive approach by utilizing KDE in conjunction with MSE or SSIM to effectively address the challenge of contaminated training data. In addition, we provide quantitative visualization of localized anomalies. Our goal is to assist researchers in comprehending the basic concepts behind autoencoder-based visual anomaly detection, to design autoencoder models with high classification performance, and to localize defect areas on an image to find the actual defective part of the product. The following section describes ongoing research on the topic of autoencoders and anomaly detection. The materials and methods are introduced in Section 3. Section 4 covers the experiments and discusses their results. Finally, Section 5 is a summary of our work in which we also discuss future research directions.

## 2. Related Work

Autoencoders were first introduced by David E. Rumelhart et al. in their paper titled “Learning internal representations by error propagation” [6] as a deep learning model that can be trained to reconstruct its input data. Based on [7], autoencoders were originally designed for dimensionality reduction and encoding the input to latent representations [8]. Their capability for anomaly detection and inference from their training data distribution was discovered later in [9]. Jie Yang et al. have collected and categorized a considerable amount of research on anomaly detection in a 2021 survey [5]. In that survey, the authors comprehensively studied the classical methods along with deep learning-based methods for visual anomaly detection in the literature. They described each method’s principles, assumptions, advantages, and disadvantages. Methods like density estimation, one-class classification, autoencoders, and GANs have been thoroughly researched, and the results have been presented. The authors emphasize leveraging both the distribution of representations in latent spaces and the reconstruction errors of autoencoders, which can further improve anomaly detection performance. Yet, the details of the performance gain have not been discussed. Research by Lara Beggel et al. [10] discussed autoencoders and their limitations. The result of autoencoder-based anomaly detection techniques is particularly susceptible to minor deviations from clean training sets. The presence of a few defective samples contaminating the training dataset causes the autoencoder to learn and reconstruct both defective and normal samples in the training phase and makes the reconstruction score incompetent [10]. That paper examined the fundamental factors that lead to traditional autoencoders’ vulnerability to anomalies in training datasets and proposed a number of crucial concepts that make autoencoder models more resistant to them, enhancing the performance of anomaly detection as a whole. That paper put forward the use of likelihood-based anomaly detection using likelihood in an autoencoder latent space. Generative methods like the variational autoencoders proposed in [11,12] and generative adversarial networks [13] are used for defect detection across much work in the literature, yielding better results [14,15,16,17]. Nevertheless, despite these improvements, autoencoders are still effective methods in anomaly detection for low-texture image data like industrial thermal images. Deep neural network training can be accelerated, and its generalization can be improved with the use of normalization techniques. We have benefited from [18], which discusses normalization techniques in the context of deep learning. In addition, ref. [19] discusses the sensitivity of outlier and anomaly detection methods to different types of normalizations.

The structural similarity index measure (SSIM), proposed in [20], analyzes the inter-dependencies among neighboring pixels that are spatially close to each other. When intensity values remain nearly constant, the authors of that paper argue that utilizing a reconstruction error fails to identify defective regions that have undergone visual modification. This prevents the reconstruction-error-based approach from being applied to complex real-world scenarios, so they proposed using a perception-based loss function based on structural similarity that inspects inter-dependencies between pixels that are spatially close in a given region, instead of simply comparing pixel values. The authors claimed that this method achieves significant performance gains. The structural similarity index measure (SSIM) was then introduced by [21]. In their work [22], Patrik Feeny et al. went over how deep autoencoders’ pixel-wise reconstruction errors are frequently used for localizing and detecting visual novelty. According to their explanation, high-error pixels point to the parts of the input image that are unfamiliar and so more likely to contain novelty. The authors of [20] discussed localizing anomalies by creating residual maps based on the pixel-wise $l^{2}$ distance and the perceptual similarity metric based on SSIM. They claimed that the residual map produced by SSIM accurately locates defects by putting more importance on the visually noticeable differences introduced by the model. Flex Meissen et al. [23] trained an autoencoder model using SSIM loss, and their method significantly increased the performance on two medical datasets for brain MRIs and localized the anomalies on the images. The authors of that paper believed that it is necessary to take a similar approach to localize defective areas in industrial thermal images efficiently.

Our approach considers the likelihood model introduced in [10], SSIM [20,21], and reconstruction errors based on MSE to define anomaly scores and to tackle challenges in the defect detection of industrial thermal images. Furthermore, applying the concepts in [20,22,23] on thermal image datasets, a parameterized method for localization is introduced in this article to identify defective areas in thermal images and consequently on industrial products.

## 3. Materials and Methods

After the characteristics of the datasets and used data pipeline are introduced, the research method is discussed in detail. The approach consists of two phases. First, the proposed autoencoder models are developed and trained in the training phase. Then, in the test phase, anomaly score outputs are used for classification performance analyses.

### 3.1. Dataset and Pipeline

**Figure 1:**
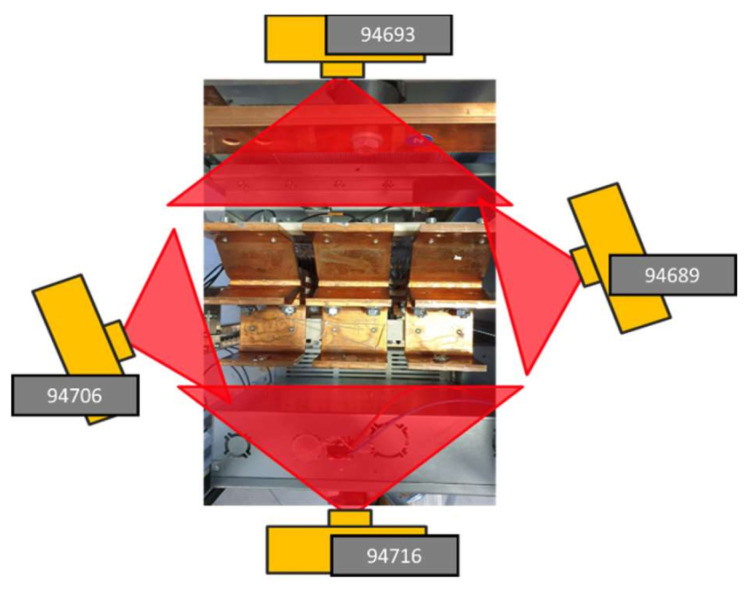
Position of four cameras monitoring switchgear equipment [4].

To evaluate our anomaly detection approaches, we used 32 × 32-sized thermal images of switchgear equipment. The images were captured using four infrared cameras monitoring low-voltage switchgear busbar cable connections (Figure 1). The cameras recorded a healthy baseline for a period of time; then, several faults were introduced to the switchgear. The faults were simply different loose cable connections that create notable intense hotspots on the thermal images.

**Figure 2:**
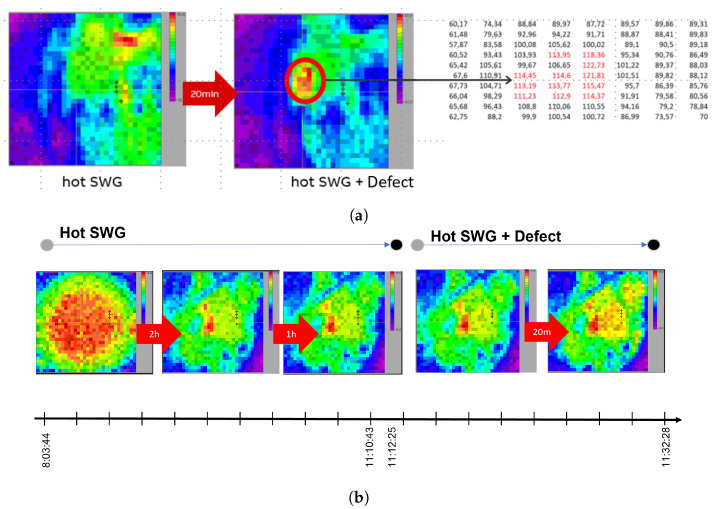
(**a**) Healthy and defective samples with relative pixel values. Defective pixel values exceeded a threshold highlighted (**b**). Thermal image transition from healthy to defective within 4 h experiment.

Datasets are image sequences captured during equipment runtime. The cameras were pointed in the direction of the switchgear busbar and captured thermal images within approximately 4 h of the test time. After three hours of recording baseline healthy images, defects were introduced. Consequently, the temperature changes in the healthy data emerged on the images within a 20–30 min time window (Figure 2). Four scenarios were defined according to the type of loose connections, resulting in four different defective datasets: *FaultL1K1*, *FaultL1K1K5*, *FaultL2K3*, and *FaultL2K6-7*. In this context, “*L*” corresponds to the phase number, and “*K*” denotes the contact number on the busbar. To exemplify, *FaultL1K1* is a loose connection in phase 1 of the first contact on the busbar accordingly. After labeling the healthy and defective images, a small amount of defective labeled data became available, which led us to the classic problem of imbalanced data in the anomaly detection problem.

There were few samples of defective images inside the healthy datasets, which made the training data clean. This was the case for all cameras except 94693, which was contaminated for experimental purposes.

It was necessary to split the data for the training and testing phases. The testing phase had two sub-phases: threshold determination and classification. To achieve the best results, we needed to isolate the data to avoid the models from being exposed to data repeatedly within the mentioned phases. The datasets were split according to a schema: 80% of healthy images were used for training $X_{train}$ and 20% was used for classification $X_{healthy-class}$. Three defective datasets were combined, shuffled, and used for threshold determination $X_{defect-test}=FaultL1K1+FaultL1K1K5+FaultL2K3$. One defective dataset, FaultL2K6-7, was used for classification $X_{defect-class}$. We determined the thresholds with three types of defective images and tested the threshold performance on the unseen datasets, including $X_{healthy-class}$ labeled as $y_{healthy-class}$ and $X_{defect-class}$ labeled as $y_{defect-class}$. In our experiment, we utilized the median–IQR and min–max normalization methods, which are robust to outliers [19]. As a result, min–max normalization on thermal images resulted in better performances for all approaches.

**Figure 3:**
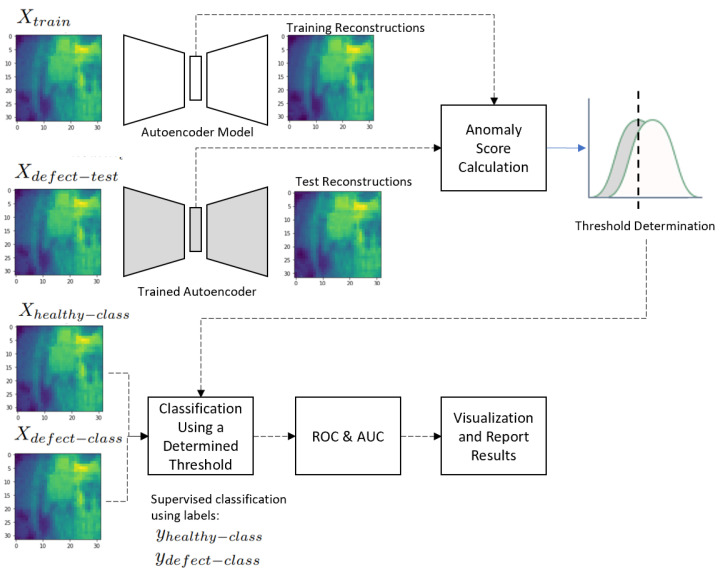
Data pipeline of the anomaly detection network.

Figure 3 describes the data pipeline used to evaluate our anomaly detection network. In all the research approaches, we inserted the data into the pipeline as follows:

- In the training phase, a model was trained with $X_{train}$ with its respective loss functions.
- In the test phase, the anomaly score distributions for $X_{train}$ and $X_{defect-test}$ were visualized and the threshold for classification was determined $Threshold=max(A\left(X_{train}\right))$.
- $X_{healthy-class}$ and $X_{defect-class}$ were combined and used for supervised classification using the data labels $y_{healthy-class}$ and $y_{defect-class}$.
- The classification performance measures with $Threshold=max(A\left(X_{train}\right))$ were calculated.
- The false-positive rate and the true-positive rate for 30 thresholds were determined; a receiver operating characteristic (ROC) curve was drawn; and accordingly, the area under the curve (AUC) was calculated.
- Result tables were prepared with the performance measures, ROC, and the AUC results.
- A visualization of the healthy and defective samples was created, along with residual maps for quantitative analysis.

### 3.2. Anomaly Detection Using Autoencoders

Anomaly detection is commonly characterized as follows: Having a dataset including many normal samples *x* and few anomalous samples $\hat{x}$, model *M* is trained to learn the distribution of the normal observations p(x) and, during the test, detects anomalous samples $\hat{x}$ by producing an anomaly score A(x), where *x* is a predetermined test sample. Since model *M* is trained to minimize/maximize (depending on the type of anomaly score) this score through optimization, A(x) can identify hidden anomalies as being out of alignment with p(x) [14].

An autoencoder comprises encoder and decoder functions implemented as a multi-layer neural network. The encoder function maps the inputs to a lower dimension *z*, known as latent space f:x→z. The decoder reconstructs the input *x* from the latent representations *z* and outputs $x^{\prime }$, $g:z\to x^{\prime }$. This network is optimized to minimize the loss function $L(x,x^{\prime })$. This loss function has to be defined in a way that represents the difference between *x* and $x^{\prime }$. Our focus in this paper was the mean squared error and structural similarity index measure (SSIM) as loss functions. Anomaly detection in a test dataset was carried out by classifying the observations *w* with A(w:g(f(w)))>T, where *T* is the decision threshold. *T* was chosen based on the distance between the distribution of anomaly scores of known defective samples and the training set $X_{train}$ [10]. We studied and conducted experiments based on three different anomaly scores: MSE, SSIM, and kernel density estimation. A combination of these anomaly scores can be used to boost performance. Theoretically, it was assumed that the training dataset was clean without any anomaly instances. Still, in real-world datasets, which might contain anomalous samples (as is in camera 94693), this is typically not the case. Contamination of the healthy images with defective ones impacts the performance metrics and must be reduced. The models and computation codes utilized in this study are readily accessible, as detailed in the supplementary materials section.

#### 3.2.1. Training Autoencoder

In the training phase, the proposed autoencoder models were developed based on a CNN architecture and trained with healthy thermal images optimizing two different loss functions: mean squared error (MSE) and structural similarity index measure (SSIM).

##### Mean Squared Error as Loss Function

In this approach, for scoring abnormalities, we used mean squared error $L(x,x^{\prime })$. Images that significantly differ from those seen during training will result in errors during reconstruction:

(1) $$L\left(x,x^{\prime }\right)=\sum {∥x-x^{\prime }∥}^{2}$$

The autoencoder was trained using $L(x,x^{\prime })$ or mean squared error (MSE) as a loss function. The loss function was optimized through gradient descent optimization to reach the minimum for healthy data. The outcome of training was an autoencoder reconstructing input images and output images similar to healthy instances.

##### Structural Similarity as Loss Function

SSIM was first introduced in [21]. The authors put forward a system for evaluating quality based on how structural information changes. The SSIM extracts three key features from an image: luminance, contrast, and structure. It calculates the SSIM, which ranges from −1 to +1, between two images. A value of 1 shows that the input images are identical and −1 indicates that the input images are different by far. Basically, between two image patches *a* and *b*, the structural similarity index defines a measurement of distance. The size of the patch is defined by variable *K*:

(2) $$SSIM_{\left(a,b\right)}=l{\left(a,b\right)}^{\alpha }c{\left(a,b\right)}^{\beta }s{\left(a,b\right)}^{\gamma }$$

where α, β, and γ are constants to weights *l*, *c*, and *s*. The latter weights are functions of mean, variance, and covariance of intensities:

(3) $$l\left(a,b\right)=\frac{2\mu_{a}\mu_{b}+c_{1}}{\mu_{a}^{2}\mu_{b}^{2}+c_{1}}$$

(4) $$c\left(a,b\right)=\frac{2\sigma_{a}\sigma_{b}+c_{2}}{\sigma_{a}^{2}\sigma_{b}^{2}+c_{2}}$$

(5) $$s\left(a,b\right)=\frac{2\sigma_{ab}+c_{2}}{2\sigma_{a}\sigma_{b}+c_{2}}$$

The values of the constants $c_{1}$ and $c_{2}$ are commonly adjusted to 0.01 and 0.03 to maintain numerical stability. By substituting Equations (3)–(5) in Equation (2),

(6) $$SSIM\left(a,b\right)=\frac{\left(2\mu_{a}\mu_{b}+c_{1}\right)\left(2\sigma_{ab}+c_{2}\right)}{\left(\mu_{a}^{2}+\mu_{b}^{2}+c_{1}\right)\left(\sigma_{a}^{+}\sigma_{b}^{2}+c_{2}\right)}$$

During training, the autoencoder model learns to reconstruct the same input to the output, so it maximizes the SSIM between input and output images. So, we defined a loss function as $L=1-SSIM(x,x^{\prime })$ to be minimized during optimization.

#### 3.2.2. Finding Anomalies in Test Phase

The trained autoencoders in the previous phase can thus reconstruct normal images. When the anomaly score exceeds a certain threshold during the testing phase, the image is identified as defective [10]. We fed healthy and defective data to the autoencoder; in both cases, the autoencoder tried to reconstruct the input to a healthy image, but the attempts led to different anomaly scores. Based on the autoencoder loss function, anomaly score A(x), which can identify the difference between the input and the reconstructions of the model, can be applied for threshold determination and classification of images. Considering this fact, anomaly scores based on mean squared error (MSE), kernel density estimation (KDE), and structural similarity measure (SSIM) were applied to the autoencoder model, and the results were analyzed in this research.

##### Mean Squared Error as Anomaly Score

**Figure 4:**
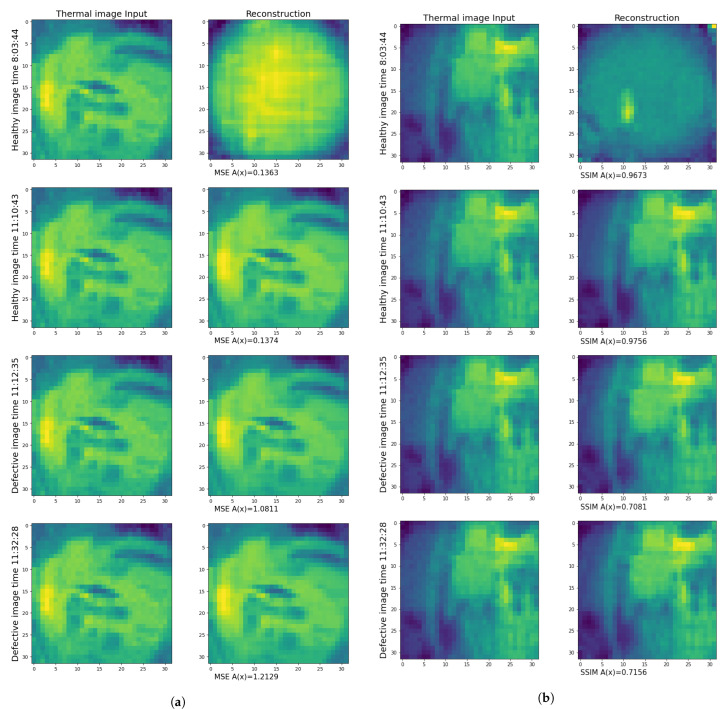
(**a**) Comparison between input and output of autoencoder with MSE loss function. Healthy image anomaly score based on MSE is higher for defect images. (**b**) Comparison between input and output of autoencoder with *L* = 1−SSIM loss function. Healthy image anomaly score based on SSIM is lower for defect images.

$A\left(x,x^{\prime }\right)=\sum {∥x-x^{\prime }∥}^{2}$ is the Euclidian distance between the input and output; Figure 4a and indicates the anomaly score difference between different instances of input data during the test.

##### Structural Similarity Index as Anomaly Score

$A\left(x\right)=SSIM\left(x,x^{\prime }\right)$ was used as an anomaly score. The autoencoder’s attempts to reconstruct the defective and healthy samples led to different SSIM. Figure 4b indicates the anomaly score difference between different instances of input data during the test.

##### Kernel Density Estimation Anomaly

The concept of likelihood in a latent space was used to define another anomaly score. Since anomalies are expected to have a low likelihood given the normal data in the training phase, the likelihood can be used as another anomaly score. We use kernel density estimation [24] to calculate the likelihood of the image belonging to the normal class. KDE for training data estimates the location of the input image vector in the latent space. We assumed that anomaly images have densities farther away from the densities of training images. Evidently, better results were obtained when likelihood and reconstruction error were combined. Regardless of loss function type during training, the KDE anomaly score can calculate the likelihood. Generally, the KDE anomaly score can be used alone in the test phase or together with other anomaly scores to improve performance.

## 4. Results and Discussion

**Figure 5:**
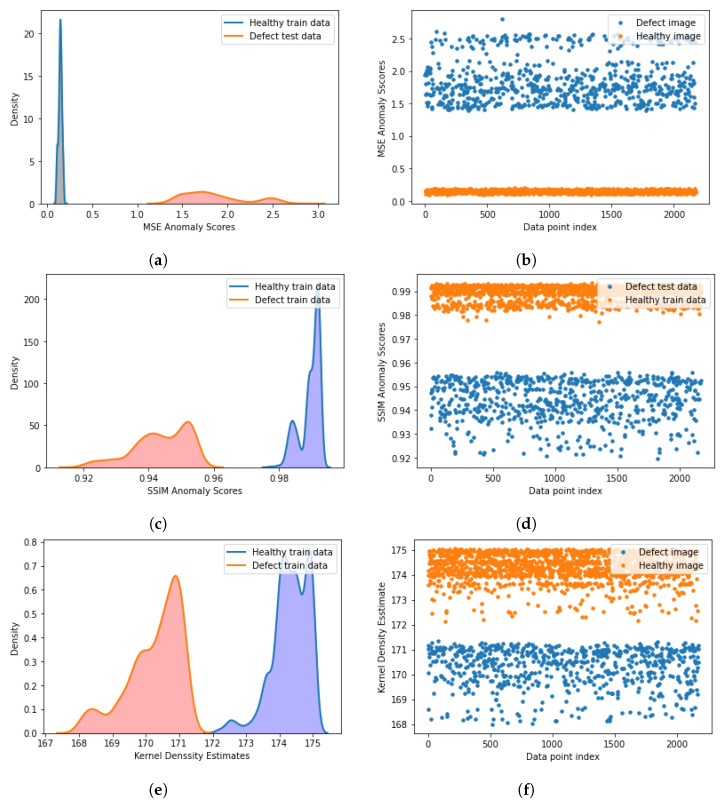
(**a**) MSE of defect and healthy samples (camera 94706). (**b**) Scatter plot of MSE anomaly scores (camera 94706). (**c**) SSIM of defect and healthy samples (camera 94706). (**d**) Scatter plot of SSIM anomaly scores (camera 94706). (**e**) Kernel density estimation of defect and healthy samples (camera 94706). (**f**) Scatter plot of Kernel density estimation anomaly scores (camera 94706).

Since the convolutional autoencoder has better performance in feature extraction [25,26,27], the convolutional autoencoder was trained with $X_{train}$. The mean squared error and SSIM loss functions were used separately for optimization. In the test phase, MSE, SSIM, and kernel density estimation distributions for $X_{train}$ and anomaly samples were used to determine *T* (Figure 5). Typical choices, based on the type of anomaly score, are the maximum or minimum of A(x) [10].

Figure 5 indicates the separation of anomaly scores for train healthy and defect test data. For SSIM and KDE, the thresholds have to be determined as $T=minA(x:x^{\prime })$.

**Figure 6:**
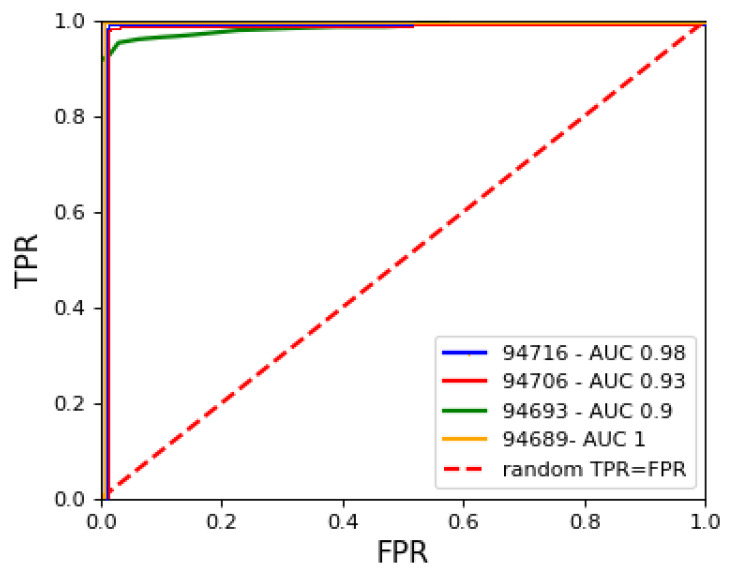
ROC curves for classification based on MSE scores. Poor performance of camera 94693 is visible.

We present the results based on the classification measures along with the area under the ROC curve (AUC). The true-positive rate values and false-positive rate values were calculated for 30 different thresholds, which consequently produced the ROC. Figure 6 presents ROC curves corresponding to four datasets.

**Figure 7:**
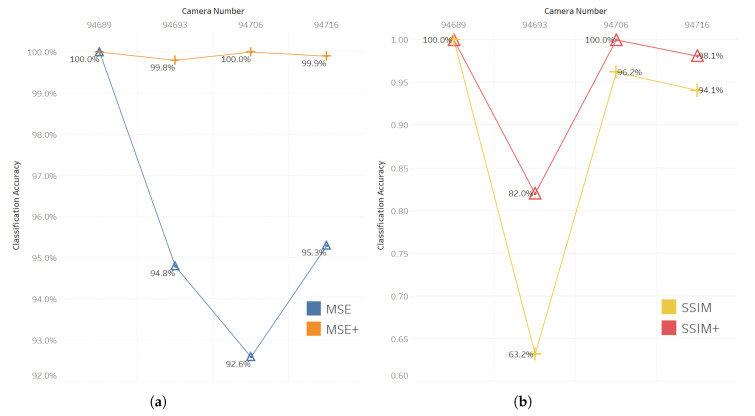
(**a**) Impact of combining KDE and MSE anomaly scores. (**b**) Impact of combining KDE and SSIM anomaly scores.

Table 1 presents the classification performance results based on the determined *T* on the anomaly scores generated using the autoencoder. The classification measures indicate high performances in all the datasets, except the contaminated dataset (camera 94693). This high accuracy is due to the nature of thermal images and can be reduced depending on the texture of the input images. Nevertheless, the SSIM outperforms the MSE anomaly score in all cases. For performance improvement, we combined the MSE and SSIM thresholds with KDE thresholds MSE+ and SSIM+. To conduct a comparative analysis, we compared the results of MSE and SSIM as the baseline approaches and further evaluated the enhanced performance achieved using the MSE+ and SSIM+ thresholds. Figure 7 displays the amount of improvement in accuracy.

**Figure 8:**
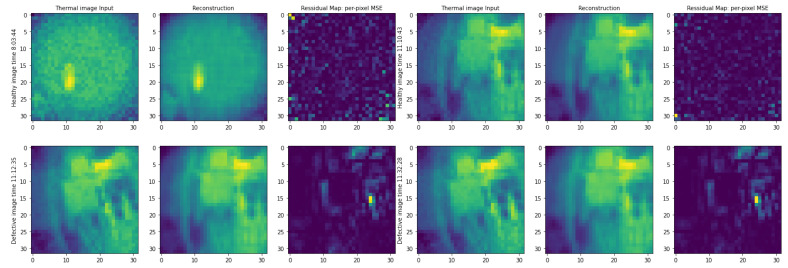
Localization of defects on the defect images using per-pixel MSE anomaly score. Defective pixels appear gradually in the defect images taken within a 20 min defect duration. The presence of yellow pixels within the residual maps signifies the specific location of defects.

We used the per-pixel reconstruction error to locate the anomalies and to localize the defective pixels on the images. We expect that pixels with high anomaly scores would locate the parts of the input image that are unfamiliar to the autoencoder and are consequently more likely to be defective [22]. In Figure 8, the MSE anomaly score is calculated for each pixel of the input and reconstructed image to generate the residual maps.

Considering the nature of SSIM, which requires a region to find inter-dependencies between pixels, measuring pixel-wise scores involves defining a region. Then, SSIM scores are calculated using Equation (6) within this defined region, called a patch. This means, depending on the patch size, we divide the spatial dimensions of input and reconstructed image to *n* patches. We propose using a parameter such as patch size to examine the quantitative performance localization by changing the patch size.

**Figure 9:**
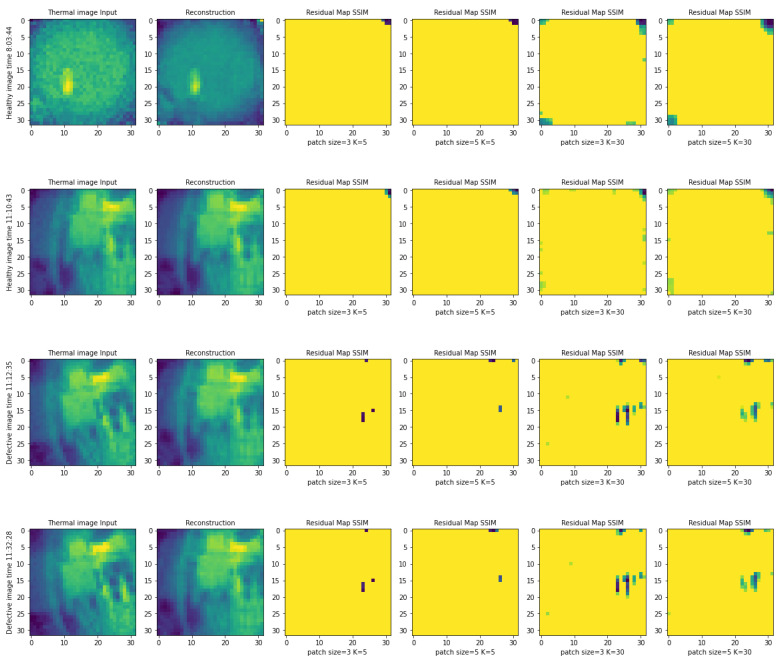
Localization of defects on the defect images using per-pixel SSIM anomaly score *k* = 5, *k* = 30, and patch size = 3, patch size = 5.

The output of patch-wise score calculation is a 32 × 32 array with values in the range of [−1,1]. Since a lower SSIM score indicates less similarity between pixels inside patches, the smallest values in the output array are the most defective parts. We introduce another parameter *k*, which is the number of smallest values needed to form a residual map. The higher *k* is, the more defective pixels are localized. Figure 9 shows the experiment results on healthy and defect images with different *k* and patch sizes.

## 5. Conclusions and Future Work

In this paper, we proposed new methods to improve the performance of autoencoder-based defect detection methods using thermal image datasets and to overcome related challenges inflicted by the sequence-based nature of the images and the smooth transition of the images through the dataset. Our research results show that our approach helps to overcome these challenges. Through our analysis, we proved that, in general, the SSIM outperforms the MSE score slightly in all cases. This is due to the nature of the SSIM, which examines structural information in image regions. Nevertheless, combining anomaly scores with KDE improves accuracy. This strongly suggests that autoencoder latent space distribution provides valuable data for the anomaly detection task. Introducing the MSE+ and SSIM+ methods, we discussed that the use of the KDE anomaly score boosts performance significantly when healthy training data are contaminated. By quantitatively analyzing localized defects in MSE and SSIM residual maps, we compared localization with the MSE and SSIM anomaly scores. Furthermore, by introducing two parameters, *k* and patch size, localization with SSIM puts forward a more extensible method to detect defects on input images.

Although the goal of this article has been accomplished, there are plenty of methods left for the future to be applied to improve the results and to tackle the challenges of defect detection in industrial thermal images. One area of future work is utilizing variational autoencoders. A variational autoencoder offers a probability measure as an anomaly score as opposed to a reconstruction error, which is a benefit over an autoencoder. Probabilities are more principled and reliable than the anomaly scores discussed in this paper [11]. Using a similar methodology in this article, the KL divergence between the Gaussian distribution and the actual distribution of a variational autoencoder’s latent space codings can be applied to find better anomaly scores. Moreover, the use of saliency maps for the localization of defective areas could be investigated. The method is proposed in [22], and we can expect to improve the results when localizing defects on industrial thermal images.

## Figures and Tables

**Table 1:** Classification performance results.

Camera	Anomaly Score	Accuracy	Precision	Recall	AUC
94716	MSE	95.33%	100.00%	98.00%	0.98
	SSIM	94.12%	94.37%	98.45%	0.98
	KDE	99.95%	100.00%	99.90%	1.00
94706	MSE	92.57%	87.07%	100.00%	0.93
	SSIM	96.20%	98.90%	99.79%	1.00
	KDE	100.00%	100.00%	100.00%	1.00
94693	MSE	94.80%	97.00%	98.80%	0.90
	SSIM	63.18%	57.60%	99.90%	0.63
	KDE	71.29%	63.52%	100.00%	0.71
94689	MSE	100.00%	100.00%	100.00%	1.00
	SSIM	100.00%	100.00%	100.00%	1.00
	KDE	100.00%	100.00%	100.00%	1.00

## Data Availability

Restrictions apply to the availability of thermal image data used in this research. The data were obtained from ABB Corporate Research Center Germany.

## Supplementary Materials

Models and computation codes are accessible through the GitHub repository at the following URL: https://github.com/Rrschch-6/Visual-Anomaly-Detection-in-industrial-thermal-images.
